# Epstein-Barr virus genome packaging factors accumulate in BMRF1-cores within viral replication compartments

**DOI:** 10.1371/journal.pone.0222519

**Published:** 2019-09-13

**Authors:** Atsuko Sugimoto, Yoriko Yamashita, Teru Kanda, Takayuki Murata, Tatsuya Tsurumi

**Affiliations:** 1 Division of Virology, Aichi Cancer Center Research Institute, Chikusa-ku, Nagoya, Japan; 2 Department of Virology, Nagoya University Graduate School of Medicine, Nagoya University, Showa-ku, Nagoya, Japan; 3 Department of Virology and Parasitology, Fujita Health University, School of Medicine, Toyoake, Japan; 4 Department of Experimental Pathology and Tumor Biology, Nagoya City University Graduate School of Medical Sciences, Nagoya, Japan; 5 Division of Microbiology, Faculty of Medicine, Tohoku Medical and Pharmaceutical University, Sendai, Japan; University of Nebraska-Lincoln, UNITED STATES

## Abstract

Productive replication of Epstein-Barr virus (EBV) during the lytic cycle occurs in discrete sites within nuclei, termed replication compartments. We previously proposed that replication compartments consist of two subnuclear domains: “ongoing replication foci” and “BMRF1-cores”. Viral genome replication takes place in ongoing replication foci, which are enriched with viral replication proteins, such as BALF5 and BALF2. Amplified DNA and BMRF1 protein accumulate in BMRF1-cores, which are surrounded by ongoing replication foci. We here determined the locations of procapsid and genome-packaging proteins of EBV via three-dimensional (3D) surface reconstruction and correlative fluorescence microscopy-electron microscopy (FM-EM). The results revealed that viral factors required for DNA packaging, such as BGLF1, BVRF1, and BFLF1 proteins, are located in the innermost subdomains of the BMRF1-cores. In contrast, capsid structural proteins, such as BBRF1, BORF1, BDLF1, and BVRF2, were found both outside and inside the BMRF1-cores. Based on these observations, we propose a model in which viral procapsids are assembled outside the BMRF1-cores and subsequently migrate therein, where viral DNA encapsidation occurs. To our knowledge, this is the first report describing capsid assembly sites in relation to EBV replication compartments.

## Introduction

Epstein-Barr virus (EBV) is a human lymphotropic virus that belongs to gamma-herpesvirus group. It is an enveloped virus with a linear double-stranded DNA genome of approximately 172 kb [1]. In most cases, EBV infection occurs during childhood without obvious symptoms and establishes a latent lifelong infection. However, in some cases EBV causes infectious mononucleosis and several types of cancers, such as Burkitt lymphoma and nasopharyngeal carcinoma.

EBV can be reactivated and execute lytic infection, which is an active state that eventually results in the production of progeny virus. Although it is not clear how and when the virus is reactivated *in vivo*, reactivation can be induced in cell culture by chemical or biological reagents, such as phorbol ester, histone deacetylase inhibitor, and tumor growth factor-β [2]. These reagents first induce the expression of the viral immediate-early genes, BZLF1 and BRLF1, which in turn induce the expression of early genes, including many that encode factors for viral DNA synthesis. Such early gene products include a viral DNA polymerase catalytic subunit (BALF5), a polymerase accessory factor (BMRF1), an ssDNA-binding protein (BALF2), and components of the helicase/primase complex (BBLF4-BSLF1-BBLF2/3). These factors assemble on the viral genome and trigger amplification of viral DNA in discrete nuclear domains known as replication compartments [3, 4]. On immunostaining, replication compartments appear as large globular or horseshoe-like structures, pushing host chromatin to the periphery of the nucleus [3]. Next, viral late genes are expressed, most of which encode viral structural proteins, such as capsid proteins and glycoproteins. These structural proteins assemble in the cells, resulting in the production of progeny viruses.

We previously reported the existence of subnuclear domains within the replication compartments, which were designated as BMRF1-cores due to being highly enriched in BMRF1 [5]. BMRF1 is a nuclear protein abundantly expressed during EBV lytic infection cycle [6, 7] that binds to BALF5 to enhance polymerase processivity [8]. We showed that most BMRF1 is tightly bound to synthesized viral genomic DNA [3, 9]. Therefore, it is speculated that, in addition to enhancement of polymerase processivity, BMRF1 protects viral genomic DNA after synthesis by directly attaching to the DNA molecule. Each replication compartment can be divided into “ongoing replication foci” and “BMRF1-cores” subdomains. Pulse-chase experiments have revealed that viral DNA genomes are synthesized at the ongoing replication foci around BMRF1-cores and then transferred inward. Homologous recombinational repair (HRR) and mismatch repair (MMR) factors assemble in EBV replication compartments [9, 10]. Our three-dimensional (3D) reconstruction studies demonstrated that HRR factors mainly localize outside BMRF1-cores, where *de novo* synthesis of viral DNA takes place, whereas MMR factors were found predominantly inside. These observations led us to speculate that viral genomic DNA synthesis is coupled with HRR outside BMRF1-cores, and subsequently with MMR inside the cores, thus presumably contributing to quality control of replicated viral genomes. We also demonstrated that BMRF1-cores spatially separate early and late gene transcription [11]. Late gene mRNAs were located inside the BMRF1-cores, while early gene mRNAs were located mainly outside, the BMRF1 cores.

Herpesviruses assemble icosahedral capsid structures and encapsidate the viral genome in the nucleus. The molecular mechanisms of herpes simplex virus type 1 (HSV-1) capsid completion have been studied extensively [12]. Based on their amino acid sequence homology with HSV-1 capsid proteins, the following are assumed to be EBV capsid proteins: BcLF1 (major capsid protein), BORF1 (triplex 1 protein), BDLF1 (triplex 2 protein), BdRF1 (scaffold protein), BVRF2 (protease) BFRF3 (small capsid protein), and BBRF1 (portal protein) [13, 14] (Table 1). The capsid is composed primarily of the major capsid protein, organized as hexameric and pentameric capsomers known as hexons and pentons, respectively [15–17]. Capsomers are linked by a triplex structure (heterotrimers formed by a single molecule triplex 1 protein and two copies of triplex 2) that serve to stabilize the procapsid and capsid [18, 19]. In addition, capsomers associate with small capsid proteins which bind to the tips of hexons [16, 20]. Preformed capsids are initially assembled with internal scaffold proteins, which are processed by scaffold-associated protease [21, 22]. Subsequently, DNA packaging proteins are required for capsid maturation, or encapsidation [12, 23–25]. Based on their homologies with HSV-1, BVRF1, BGLF1, BFLF1, and BGRF1 are thought to be EBV packaging proteins, although the nature and functions of EBV packaging factors are unclear (Table 1).

**Table 1:** EBV capsid genes and their homologs in HSV.

EBV gene	Function	Homolog gene in HSV
BcLF1	Major capsid	UL19
BORF1	Triplex 1	UL38
BDLF1	Triplex 2	UL18
BdRF1	Scaffold	UL26.5
BVRF2	Protease	UL26
BFRF3	Small capsid	UL35
BBRF1	Portal protein	UL6
BDRF1	Minor capsid/Packaging	UL15
BFLF1	Packaging	UL32
BVRF1	Minor capsid/Packaging	UL25
BGLF1	Minor capsid/Packaging	UL17

Here, we examined the spatial arrangement of the sites of capsid maturation and encapsidation in relation to EBV replication compartments via 3D surface reconstruction using confocal laser scanning microscopy and correlative fluorescence microscopy-electron microscopy (FM-EM). DNA packaging factors were located inside the viral DNA storeroom within the BMRF1-cores, while capsid structural proteins were located both outside and inside the cores. Based on these observations, we speculate that capsid maturation and DNA packaging occur within the BMRF1-cores, whereas procapsid assembly occurs outside. This is the first report on the sites of procapsid assembly and viral genome packaging in relation to viral replication compartments.

## Materials and methods

### Cell culture

Tet-BZLF1/B95-8 cells [26] were maintained in RPMI 1640 medium supplemented with tetracycline-free fetal calf serum (Clontech), puromycin, and hygromycin B. B95-8 cells were cultured in RPMI 1640 medium supplemented with fetal calf serum (Sigma). To induce lytic replication, doxycycline, a derivative of tetracycline, was added to the medium.

### Construction of epitope tagged expression plasmids

The EBV open reading frames (ORFs) encoding BORF1, BDLF1, BdRF1, BVRF2, BBRF1, BFLF1, BVRF1, and BGLF1 were amplified by PCR from the genome of EBV B95-8 strain. The PCR primer pairs used to amplify the ORFs are listed in Table 2. The PCR products were cloned in pcDNA3 as a *Hind*III-*Eco*RI fragment, with the exception of BORF1, BDLF1, and BVRF1, which were cloned as *Hind*III-*Bam*HI (for BORF1 and BDLF1) and *Bam*HI-*Eco*RI (for BVRF1) fragments, respectively. The sequences of the epitope-tagged ORFs were verified by DNA sequencing.

**Table 2:** Primer sequences used in cloning of EBV capsid genes.

Primer name	Sequence
HA-BORF1-F / *Hind*III	CGCGAAGCTTGCCACC**ATG***TATCCATATGACGTTCCAGATTACGCT*AAGGTCCAGGGGTCCGTCGA
BORF1-R / *Bam*HI	CGCGGGATCCCTAGAGAATCACCTCCCAGT
Flag-BDLF1-F / *Hind*III	CGCGAAGCTTGCCACC**ATG***GACTACAAGGACGACGATGACAAG*GATTTGAAAGTGGTAGTGTC
BDLF1-R / *Bam*HI	CGCGGGATCCTTATCTTAACCAGCAAGTGG
Myc-BdRF1-F / *Hind*III	CGCGAAGCTTGCCACC**ATG***GAGCAGAAGCTGATCAGCGAGGAGGACCTG*CTATCAGGTAACGCAGGAGA
BdRF1-R / *Eco*RI	CGCGGAATTCTCAAGCCACGCGTTTATTCA
HA-BVRF2-F / *Hind*III	CGCGAAGCTTGCCACC**ATG***TATCCATATGACGTTCCAGATTACGCT*GTGCAGGCACCGTCTGTATA
BVRF2-R / *Eco*RI	CGCGGAATTCTCAAGCCACGCGTTTATTCA
HA-BBRF1-F / *Hind*III	CGCGAAGCTTGCCACC**ATG***TATCCATATGACGTTCCAGATTACGCT*TTCAACATGAACGTGGACGA
BBRF1-R / *Eco*RI	CGCGGAATTCTTAACTTCCAGCACCAGGCG
Myc-BFLF1-F / *Hind*III	CGCGAAGCTTGCCACC**ATG***GAGCAGAAGCTGATCAGCGAGGAGGACCTG*GCTCACAAAGTCACGAGCGC
BFLF1-R / *Eco*RI	CGCGGAATTCTCACACGTAGACCTGGGAAG
Flag-BVRF1-F / *Bam*HI	CGCGGGATCCGCCACC**ATG***GACTACAAGGACGACGATGACAAG*GCGCTCTCCGGGCACGTCTT
BVRF1-R / *Eco*RI	CGCGGAATTCTCAGCTTGGGGCCACCGGGG
Myc-BGLF1-F / *Hind*III	CGCGAAGCTTGCCACC**ATG***GAGCAGAAGCTGATCAGCGAGGAGGACCTG*GATGTCCACATTGACAACCA
BGLF1-R / *Eco*RI	CGCGGAATTCCTACTGTTCGCGGCGGATGC

### Transfection

Tet-BZLF1/B95-8 cells were transfected with 2μg of expression plasmids per six-well plate by electroporation using the Neon system (Thermo Fisher Scientific). After transfection, doxycycline was added to the culture medium to a final concentration of 4 μg/mL to induce EBV lytic replication. The cells were incubated at 37°C for 24 h before harvesting.

#### Antibodies

An anti-EBV EA-D-p52/50 (BMRF1) mouse monoclonal antibody (R3) was purchased from Chemicon. An anti-5-bromo-2’-deoxcyuridine (BrdU) rat monoclonal antibody (BU1/75, ICR1) (Abcam) was used to detect 5-chloro-2’-deoxcyuridine (CldU)-labeled DNA. Anti-EBV p18 goat polyclonal antibodies were purchased from Thermo Scientific. Mouse monoclonal anti-HA (12CA5), rabbit anti-FLAG, and rabbit anti-Myc antibodies were obtained from Roche, Sigma-Aldrich, and Cell Signaling, respectively. Secondary IgG antibodies conjugated with Alexa 488, 594, or 647 and a Zenon Mouse IgG Labeling Kit (Alexa 488) were purchased from Molecular Probes.

### Immunofluorescence analysis

All staining procedures were carried out as described previously [5]. Briefly, cells were treated with 0.5% Triton X-100/mCSK buffer and fixed with 70% ethanol, permeabilized with 0.5% Triton X-100 in phosphate-buffered saline (PBS), blocked in 10% normal goat serum (or fetal bovine serum for BFRF3 staining), and incubated with the primary antibodies. After washing, the samples were incubated with secondary antibodies conjugated to Alexa Fluor 488, 594, and 680. Next, an anti-BMRF1 antibody directly labeled with Alexa Flour 488 using a Zenon Tricolor Mouse IgG1 Labeling Kit (Molecular Probes) was applied, followed by washing with PBS. Slides were mounted in ProLong Gold Antifade reagent with 4’6-diamidino-2-phenylindole (DAPI) (Molecular Probes) and analyzed by using an LSM510 Meta microscope (Carl Zeiss MicroImaging, Inc.).

### Pulse-chase experiments

Pulse-chase labeling was performed as described previously [5]. Briefly, Tet-BZLF1/B95-8 cells were induced by doxycycline for 24 h and incubated with 10 μM CldU for 10 min. After extensive washing, the medium was replaced with that containing IdU to inhibit CldU incorporation into newly synthesized DNA and incubated for 1 h. Next, the cells were treated with 0.5% Triton X-100/mCSK buffer, fixed, and then treated with 2 N HCl containing 0.5% Triton X-100 to expose the incorporated CldU residues before blocking. The cells were washed twice with PBS and neutralized with 0.1 M sodium tetraborate (pH 9.0) for 5 min prior to immunofluorescence analysis.

### Three-dimensional reconstruction by confocal laser scanning microscopy

Serial images (n = 50–100) were captured using a Mete confocal laser scanning microscope (CLSM; Carl Zeiss). The images were assembled and 3D reconstruction was performed using Imaris software (Carl Zeiss).

### Correlative FM-EM and EM

Correlative FM-EM allows individual cells to be examined by both FM and EM. Tet-BZLF1/B95-8 cells were cultured on gridded 35-mm glass-bottom dishes (Mat Tek) coated with 1% gelatin in RPMI 1640 medium. To induce lytic EBV replication, doxycycline was added to the medium. After 24 h, cells on the grid were fixed with 4% paraformaldehyde solution in phosphate buffer (PB), stained with specific antibodies, and examined by CLSM. The same specimens were postfixed with 1% osmium tetroxide and 0.5% potassium ferrocyanide in PB for 1 h on ice, washed with distilled water (three times for 1 min each), dehydrated in an ethanol series (50, 70, 90 and 100% for 5 min each), and embedded in Epon-812 (TAAB Laboratories) at 65°C for 48 h. Ultrathin sections of cells were stained with saturated uranyl acetate and Reynold’s lead citrate solution. Electron micrographs were taken using a JEM-1400EX transmission electron microscope (JEOL). For EM, pcDNA-BZLF1 was transfected by electroporation into B95-8 cells to induce the lytic phase. The cells were harvested 24 h after transfection. After fixation, dehydration, and embedding, electron microscopic analysis was performed by using a JEM-1200EX transmission electron microscope at 80kV (JEOL).

## Results and discussion

### DNA packaging factors localize within the BMRF1-core

We previously reported that BMRF1-rich structures, known as BMRF1-cores, exist within viral replication compartments, and that viral DNA genomes are synthesized outside the BMRF1-cores and then translocated inward [5]. Thus, the BMRF1-cores serve as the storage site for synthesized viral DNA genomes. Following viral genome replication, it is believed that procapsid assembly and DNA packaging take place somewhere within the nucleus. To gain insight into the site of capsid completion, we examined the spatial distribution of viral capsid structural proteins and DNA packaging factors relative to synthesized viral genome DNA within the cores.

**Fig 1:**
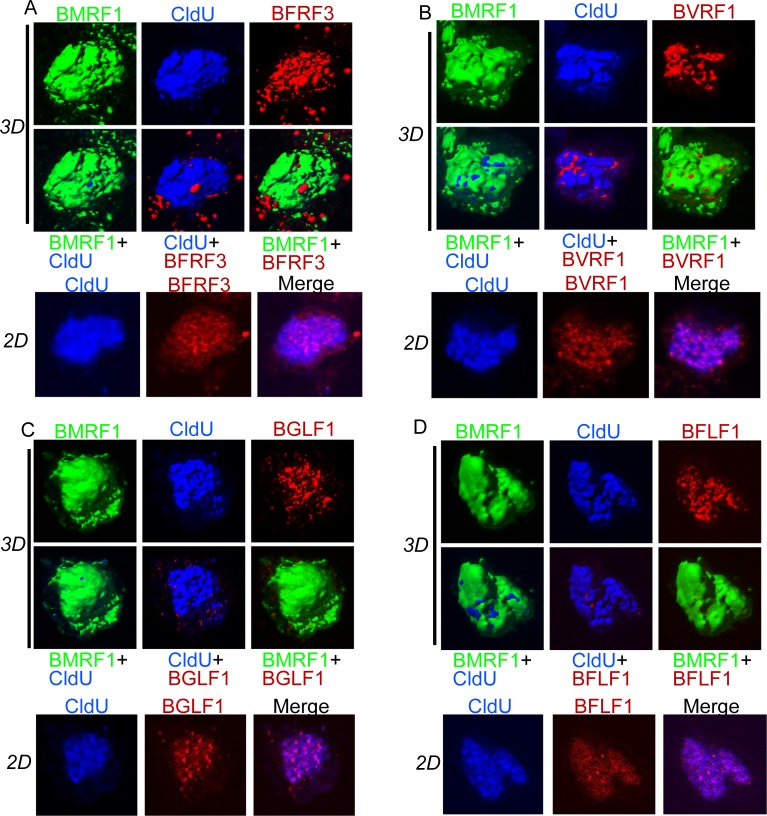
Small capsid protein and DNA packaging factors are localized inside the BMRF1-core. (A-D) Tet-BZLF1/B95-8 cells were transfected with epitope-tagged viral factors, and simultaneously treated with doxycycline to induce lytic replication. At 24 h after transfection and lytic induction, the cells were pulse-labeled with CldU for 10 min and chased for 1 h. The cells were treated with mCSK buffer, fixed, and stained with the following antibodies: (A) anti-BMRF1 (Green), anti-CldU (blue), and anti-BFRF3 (red) antibodies (B) anti-BMRF1 (Green), anti-CldU (blue), and anti-flag (red) antibodies (C) anti-BMRF1 (Green), anti-CldU (blue), and anti-Myc (red) antibodies (D) anti-BMRF1 (Green), anti-CldU (blue), and anti-myc (red) antibodies. The data are presented as three-dimensional (3D) reconstruction images (*3D*) with two-dimensional *(2D)* projection images.

Tet-BZLF1/B95-8 cells were transduced with epitope-tagged various viral proteins involved in capsid structure and DNA packaging via transfection, and treated simultaneously with doxycycline to induce lytic replication, followed by staining with epitope-specific antibodies. Exceptionally, endogenously expressed BFRF3 was stained with a specific antibody. To label the synthesized viral DNA, cells were pulsed with CldU for 10 min at 24 h post-induction, washed, and then chased for 1 h, and immunostained. In this experimental setting, CldU-labeled DNA represents viral DNA, which was synthesized within a 10-min window and transferred to the BMRF1-cores, as demonstrated previously by simultaneous fluorescence *in situ* hybridization (FISH) analysis of viral DNA [5]. As shown in 3D surface reconstruction images (Fig 1A), CldU-labeled viral genome was observed within the BMRF1-core, indicating that synthesized DNA is stored in the core.

We first examined the spatial distribution of the endogenously expressed putative EBV small capsid protein, BFRF3, relative to BMRF1 and synthesized viral DNA, by means of triple-color 3D surface reconstruction imaging (Fig 1A). BFRF3 is a homolog of the HSV VP26 (UL35) small capsid protein. During productive replication of HSV, VP26 small capsid protein is not assembled onto procapsids, instead being recruited during procapsid angularization, which may occur as viral DNA is encapsidated [27]. Thus, EBV BFRF3 is presumably recruited to the site of viral DNA encapsidation. Most of the BFRF3 was located within CldU-labeled viral DNA (Fig 1A). This result suggests that the site of EBV capsid maturation is inside the viral genome storeroom within the BMRF1-cores.

We then used the same strategy to examine the spatial distribution of exogenously expressed putative EBV DNA packaging proteins, BVRF1, BGLF1 and BFLF1 (Fig 1B–1D). DNA packaging proteins are thought to be involved in the final step of encapsidation, or capsid maturation. EBV BVRF1 is a homolog of HSV UL25, which interacts with triplex 1 proteins and major capsid proteins, and acts together with UL17 to retain packaged DNA in capsids following DNA cleavage [28]. As shown in Fig 1B, the majority of BVRF1 was surrounded by viral DNA within the BMRF1-core; a minority appeared on the core surface. EBV BGLF1 is a homolog of HSV UL17, whereas EBV BFLF1 is a homolog of HSV UL32. Although the precise functions of HSV-1 UL17 and UL32 during encapsidation are unclear, they are required for the HSV DNA cleavage/packaging reaction [25, 29, 30]. BFLF1 is believed to be involved in capsid maturation and packed DNA retention, because double knockout of BFLF1 and BFRF1A by cassette insertion resulted in the production of DNA-free virus-like particles [31]. In this study, both BGLF1 and BFLF1 were almost completely covered by viral DNAs within the BMRF1-core (Fig 1C and 1D). Thus, the putative EBV DNA packaging proteins were located inside the BMRF1-core. These results imply that capsid maturation and DNA cleavage/packaging occur inside the BMRF1-core, which contains stored viral DNA.

### Procapsid assembly might occur outside the stored viral genome

**Fig 2:**
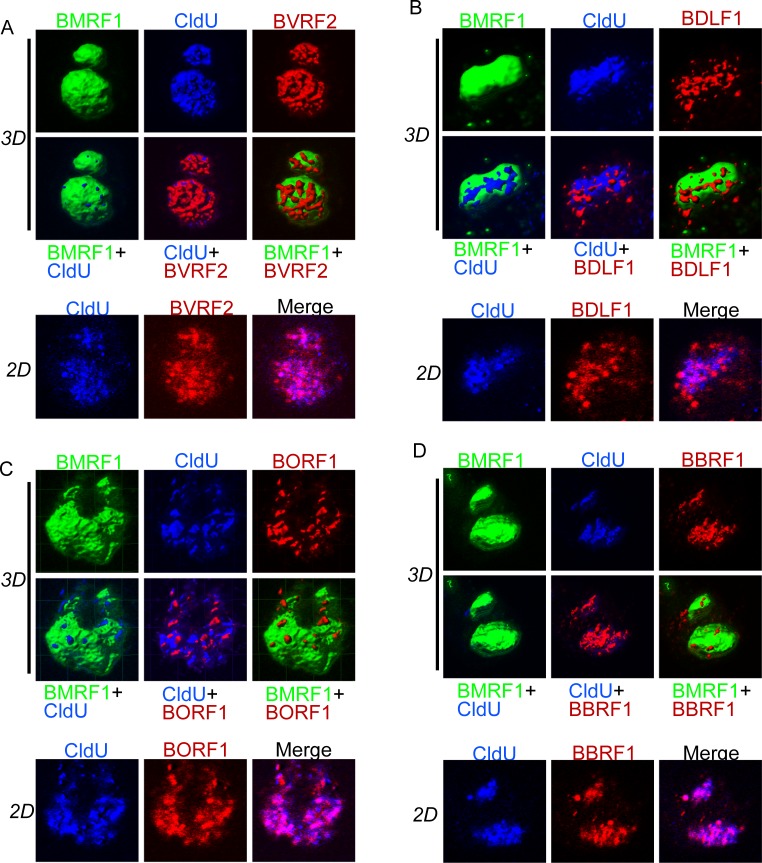
Capsid structural proteins localize outside the BMRF1-core. (A-D) The cells were processed as described in the legend to Fig 1 and stained with the following antibodies: (A) anti-BMRF1 (Green), anti-CldU (blue), and anti-HA (red) antibodies (B)anti-BMRF1 (Green), anti-CldU (blue), and anti-flag (red) antibodies (C) anti-BMRF1 (Green), anti-CldU (blue), and anti-HA (red) antibodies (D) anti-BMRF1 (Green), anti-CldU (blue), and anti-HA (red) antibodies. The data are presented as 3D reconstruction images (*3D*) with corresponding projection images *(2D)*.

Premature capsid (procapsid) assembly is the first step in capsid maturation [13]; the viral DNA genome is subsequently incorporated into the procapsid through a ring-shaped portal [31–33]. PML bodies are thought to be important for procapsid assembly, as they interact with BORF1 triplex 1 in P3HR1 cells [34]. We therefore examined the spatial distribution of the exogenously expressed viral factors that are involved in procapsid assembly. The BVRF2 protein is a protease, which assembles into the procapsid to digest scaffold proteins [13]. In contrast to the DNA packaging proteins, BVRF2 proteins were distributed both outside and inside the BMRF1-core (Fig 2A). The BDLF1 and BORF1 proteins, which are triplex proteins that constitute the procapsid shell, were similarly distributed (Fig 2B and 2C). EBV BBRF1 protein is a homolog of HSV UL6, which constitutes a dodecameric ring structure, called the portal-vertex and is loaded on early partial capsids [33, 35]. BBRF1 mainly surrounded the stored viral DNAs within the BMRF1-core (Fig 2D), although some was detected outside. Because these procapsid components also exist in mature capsids with packaged viral genomes, these proteins were observed inside the stored DNA within the BMRF1 core (Fig 2 and 2D). We speculate that procapsids are assembled outside of the subdomain where newly synthesized viral genomes are enriched, and viral genome packaging into the capsids subsequently occurs inside.

To further confirm these results, we carried out quantitative analyses (Table 3). Viral proteins required for DNA packaging, such as BGLF1, and the small capsid protein BFRF3, were mostly (86 and 73%, respectively) localized inside the BMRF1-core (Fig 1). Viral capsid structural proteins, such as BVRF2 and BDLF1, predominantly existed outside the core (91 and 87% respectively), supporting the data in Fig 2. These results reinforce our finding that empty procapsids are formed outside the BMRF1-cores, while viral DNA genome is packaged in a deeper part of the cores.

**Table 3:** Location of capsid proteins in relation to the BMRF1-cores.

	Total number of cells counted	Almost all inside BMRF1-cores (%)	At least a portion was outside BMRF1-cores		
			Mostly inside BMRF1-cores (%)	Distributed almost equally inside and outside (%)	Mostly outside BMRF1-cores (%)
BVRF2	22	2 (9)	2 (9)	13 (59)	5 (23)
BFRF3	22	16 (73)	6 (26)	0 (0)	0 (0)
BGLF1	21	18 (86)	1 (5)	2 (9)	0 (0)
BDLF1	23	3 (13)	2 (9)	5 (22)	13 (57)

### Correlative FM-EM analysis of viral replication compartments

**Fig 3:**
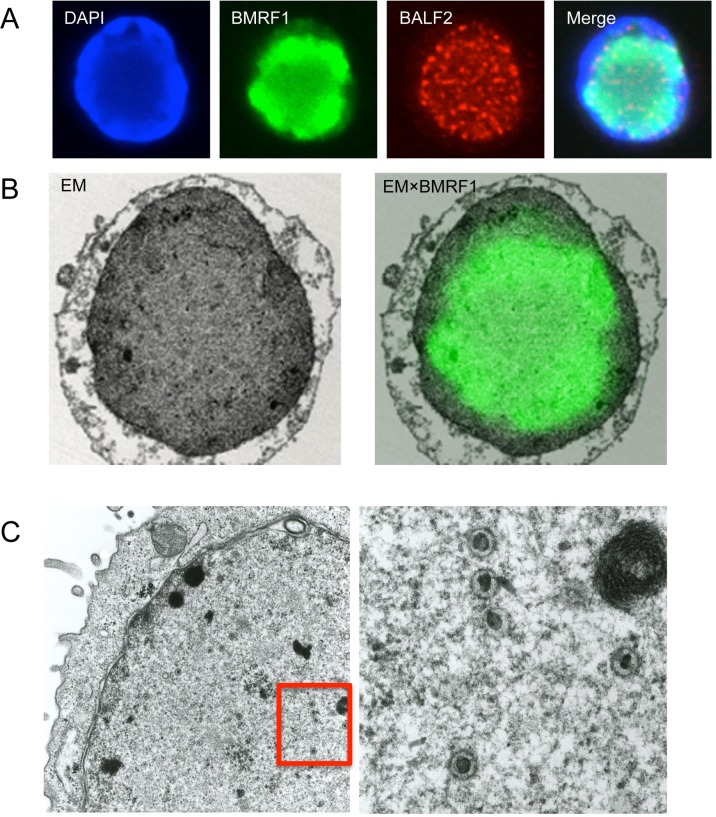
Correlative fluorescence microscopy-electron microscopy (FM-EM) and EM images of viral replication compartments. (A) Tet-BZLF1/B95-8 cells treated with doxycycline to induce the lytic cycle were fixed and stained with DAPI (blue), anti-BMRF1 (Green), and anti-BALF2 (red) antibodies. (B) The same cell in (A) was visualized using the correlative FM-EM technique (left panel). Right panel is a merged image with BMRF1 staining in (A). (C) B95-8 cells were lytically induced for 24 h and processed as described. The sample was observed by EM. The boxed area in the left panel is shown at higher magnification in the right panel. Mature viral nucleocapsids are located at the innermost part of the putative replication compartment.

We have showed that viral capsid proteins were found both inside and outside the BMRF1-cores, and packaging proteins were located predominantly inside the cores, suggesting that viral genome packaging takes place in the innermost part of BMRF1-cores. To verify this hypothesis, we examined if packaged nucleocapsids could be found in the BMRF1-cores by correlative FM-EM analysis (Fig 3). B95-8 cells were lytically induced and stained with BMRF1 and BALF2, along with DAPI (Fig 3A). In this typical cell, BMRF1 staining occupied most of the nucleus, pushing intense DNA staining (DAPI), which likely represents host cell chromatin, outside. As expected, BALF2 was visible as a punctate pattern mostly outside the BMRF1-core. The same cell was analyzed by EM (Fig 3B). The margin of the BMRF1-core was unclear in EM images without immunostaining, but the less dense part at the center of the nucleus appeared to be the core. In this less dense part of the nucleus, we frequently found packaged nucleocapsids with condensed black structures at their centers (Fig 3C). These results suggest that the packaging of viral genomic DNA occurs at the innermost part of the BMRF1-cores.

## Conclusions

**Fig 4:**
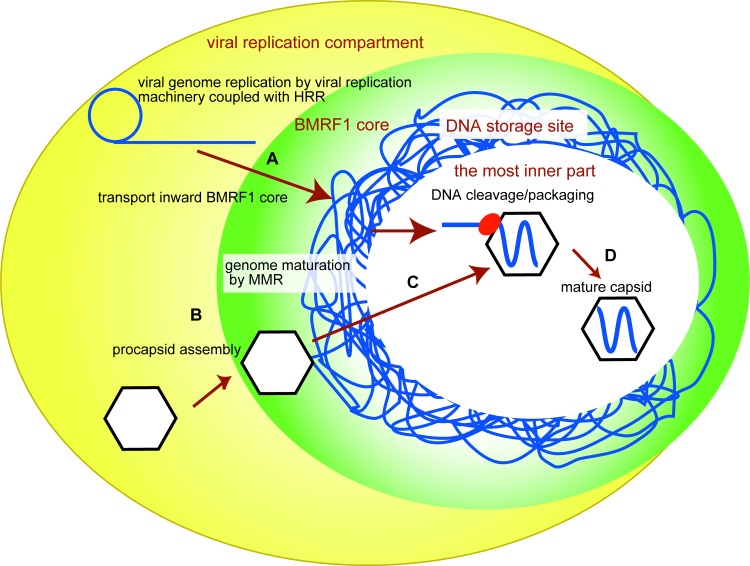
Hypothetical model of viral genome and capsid maturation. (A) Viral DNA is synthesized outside the BMRF1-core, and then transported to a DNA storage site within the BMRF1-core. (B) Procapsids (composed of capsid structural proteins, including BVRF2, BDLF1, BORF1, and BBRF1) are assembled outside the DNA storage site, which is independent of the BMRF1-core. (C) Assembled procapsids are transported inside to the DNA storage site within the BMRF1-core. (D) Cleaved unit-length viral DNA is packaged into procapsids to form mature capsids in the innermost part of the BMRF1-core. The small capsid protein BFRF3, and the DNA packaging factors BVRF1, BGLF1, and BFRF1 are involved in this process.

In a previous report, we identified a BMRF1-rich structure (BMRF1-core) within EBV replication compartments and proposed the following model. Viral DNA synthesis begins in punctate structures within the nuclei. Synthesized DNA accumulates in storerooms, i.e. BMRF1-cores. While BMRF1-cores are being enlarged, viral DNA amplification occurs, in the ongoing replication foci around the BMRF1-cores. Interestingly, ongoing replication foci outside the cores, where viral genome replication occurs, are associated with HRR factors, whereas MMR factors are co-localized with stored viral DNA in the BMRF1-cores [5]. Also, late gene mRNAs and BCRF1 protein, one of the vPIC (viral preinitiation complex) factors, were located inside the BMRF1-core, suggesting that EBV late genes are transcribed from the synthesized viral genome in the core [11]. Therefore the BMRF1-cores partition the replication compartments into inside and outside subdomains, specifying viral genome synthesis and maturation. From the present study, we propose an advanced model of the architecture of EBV replication compartments. We here suggest that the innermost part of the BMRF1 core is the site of mature capsid assembly. However, because some capsid components were observed outside of the BMRF1-cores, procapsid assembly appears to be independent of the BMRF1-cores. *In vitro* self-assembly of procapsids was demonstrated by the production of capsid proteins upon coinfection of insect cells with six viruses expressing procapsid components, as shown by EM and sedimentation assay [13]. The components could self-assemble to form procapsids after co-localizing within the replication compartments in the nuclei. Our data suggest that procapsids are assembled outside and then migrate into the storeroom where synthesized viral genome DNA awaits encapsidation. DNA packaging may occur in the innermost part of the BMRF1-core; the mechanism by which mature capsids exit the cores in unclear. Our hypothetical model of EBV genome and capsid maturation in the replication compartments is depicted in Fig 4. (A) Viral DNA is synthesized outside of, and then transported to, DNA storage sites within the BMRF1-core. (B) Procapsids are assembled outside of the DNA storage sites, which exist independent of the BMRF1-cores. (C) Assembled procapsids are transported to the DNA storage sites within the BMRF1-cores. (D) Cleaved unit-length viral DNA is packaged into procapsids to form mature capsids in the innermost part of the BMRF1-cores.

Packed DNA is free of proteins and very tightly condensed inside the capsid [12]. Therefore, BMRF1 proteins must be removed, probably by DNA packaging factors or other proteins, when DNA packaging occurs in BMRF1-cores. Our recent demonstration of the crystal structure of the BMRF1 protein showed that the molecular structure of the monomer shares structural similarity with proliferating cell nuclear antigen (PCNA) [36]. It exists mainly as a C-shaped head-to-head homodimer but also forms 50-Å-diameter tetrameric rings, which are sufficiently large to accommodate template DNA. We speculate that BMRF1 proteins bind to synthesized viral DNA through basic amino-acid residues on the concave surface, thus forming the BMRF1-core. Alternatively, BMRF1 may slide, like PCNA, on synthesized viral DNA and not be packaged into capsids. PCNA, known as a “sliding clamp”, adopts a ring-shaped trimer conformation, forming a central channel to accommodate template DNA [37]. Thus, we conclude that BMRF1 functions to protect synthesized viral DNA until it is packaged into capsids.
